# A Distinct Contractile Injection System Gene Cluster Found in a Majority of Healthy Adult Human Microbiomes

**DOI:** 10.1128/mSystems.00648-20

**Published:** 2020-07-28

**Authors:** Maria I. Rojas, Giselle S. Cavalcanti, Katelyn McNair, Sean Benler, Amanda T. Alker, Ana G. Cobián-Güemes, Melissa Giluso, Kyle Levi, Forest Rohwer, Barbara A. Bailey, Sinem Beyhan, Robert A. Edwards, Nicholas J. Shikuma

**Affiliations:** a Viral Information Institute, San Diego State University, San Diego, California, USA; b Department of Biology, San Diego State University, San Diego, California, USA; c Computational Science Research Center, San Diego State University, San Diego, California, USA; d Department of Mathematics and Statistics, San Diego State University, San Diego, California, USA; e Department of Infectious Diseases, J. Craig Venter Institute, La Jolla, California, USA; University of California, San Diego

**Keywords:** CIS, microbiome, secretion system, T6SS, bacteriophage, eCIS

## Abstract

Many commensal bacteria antagonize each other or their host by producing syringe-like secretion systems called contractile injection systems (CIS). Members of the *Bacteroidales* family have been shown to produce only one type of CIS—a contact-dependent type 6 secretion system that mediates bacterium-bacterium interactions. Here, we show that a second distinct cluster of genes from *Bacteroidales* bacteria from the human microbiome may encode yet-uncharacterized injection systems that we term *Bacteroidales* injection systems (BIS). We found that BIS genes are present in the gut microbiomes of 99% of individuals from the United States and Europe and that BIS genes are more prevalent in the gut microbiomes of healthy individuals than in those individuals suffering from inflammatory bowel disease. Gene clusters similar to that of the BIS mediate interactions between bacteria and diverse eukaryotes, like amoeba, insects, and tubeworms. Our findings highlight the ubiquity of the BIS gene cluster in the human gut and emphasize the relevance of the gut microbiome to the human host. These results warrant investigations into the structure and function of the BIS and how they might mediate interactions between *Bacteroidales* bacteria and the human host or microbiome.

**IMPORTANCE** To engage with host cells, diverse pathogenic bacteria produce syringe-like structures called contractile injection systems (CIS). CIS are evolutionarily related to the contractile tails of bacteriophages and are specialized to puncture membranes, often delivering effectors to target cells. Although CIS are key for pathogens to cause disease, paradoxically, similar injection systems have been identified within healthy human microbiome bacteria. Here, we show that gene clusters encoding a predicted CIS, which we term *Bacteroidales* injection systems (BIS), are present in the microbiomes of nearly all adult humans tested from Western countries. BIS genes are enriched within human gut microbiomes and are expressed both *in vitro* and *in vivo*. Further, a greater abundance of BIS genes is present within healthy gut microbiomes than in those humans with with inflammatory bowel disease (IBD). Our discovery provides a potentially distinct means by which our microbiome interacts with the human host or its microbiome.

## Abstract (precis)

To engage with host cells, diverse pathogenic bacteria produce syringe-like structures called contractile injection systems (CIS). CIS are evolutionarily related to the contractile tails of bacteriophages and are specialized to puncture membranes, often delivering effectors to target cells. Although CIS are key for pathogens to cause disease, paradoxically, similar injection systems have been identified within healthy human microbiome bacteria. Here, we show that gene clusters encoding a predicted CIS, which we term *Bacteroidales* injection systems (BIS), are present in the microbiomes of nearly all adult humans tested from Western countries. BIS genes are enriched within human gut microbiomes and are expressed both *in vitro* and *in vivo*. Further, a greater abundance of BIS genes is present within healthy gut microbiomes than in those humans with with inflammatory bowel disease (IBD). Our discovery provides a potentially distinct means by which our microbiome interacts with the human host or its microbiome.

## INTRODUCTION

**FIG 1:**
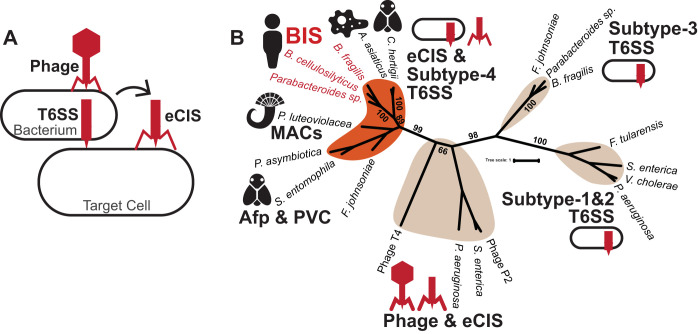
*Bacteroidales* possess a distinct contractile injection system gene cluster. (A) Contractile injection systems are related to the contractile tails of bacteriophages. There are two main types of CIS; type 6 secretion systems (T6SS) are bound to the bacterial cell membrane and act from within the producing cell, while extracellular CIS (eCIS) are released by bacterial cell lysis and bind to target cells. (B) Unrooted phylogeny of CIS sheath protein sequences. A BIS group with a known subtype 4 T6SS and eCIS (orange) are distinct from organisms with known subtype 1, subtype 2 and subtype 3 T6SS (Table S1). Bacteria with BIS identified in this study are highlighted in red. Bootstrap values are expressed in numbers of occurrences that support the phylogenetic structure out of 100, from 1,000 resampling events.

Many bacteria produce syringe-like secretion systems called contractile injection systems (CIS) that are related to the contractile tails of bacteriophages (bacterial viruses) (1, 2). CIS are composed of conserved structural elements, including a rigid inner tube surrounded by a baseplate complex and contractile sheath. Contraction of the sheath propels the inner tube through cell membranes, often delivering protein effectors to target cells (3, 4). Most CIS characterized to date, termed type 6 secretion systems (T6SS), are produced and act from within an intact bacterial cell (Fig. 1A). In contrast, extracellular CIS (eCIS) are released by bacterial cell lysis, paralleling the mechanism used by tailed phages to escape their bacterial host (5–7) (Fig. 1A). Prominent producers of CIS are members of the *Bacteroidetes* phylum (*Bacteroides* and *Parabacteroides*), which constitute 20 to 80% of the total human microbiome composition (8). To date, *Bacteroides* from the human gut have been shown to produce one type of CIS, a subtype 3 T6SS that mediates bacterium-bacterium interactions and helps them colonize the human gut (9–12).

10.1128/mSystems.00648-20.4 TABLE S1 Sequence similarity of sheath and tube proteins from representative secretion systems used to construct phylogenetic trees against BIS proteins. Download Table S1, DOCX file, 0.02 MB. Copyright © 2020 Rojas et al. 2020 Rojas et al. This content is distributed under the terms of the Creative Commons Attribution 4.0 International license.

Distinct from *Bacteroidales* subtype 3 T6SS is a different class of CIS that may have evolved independently (13). Intriguingly, all previously described examples of these distinct CIS mediate bacterium-eukaryote interactions. Three of these CIS are classified as eCIS and include (i) metamorphosis-associated contractile structures (MACs) that stimulate the metamorphosis of tubeworms (5, 14, 15), (ii) *Photorhabdus* virulence cassettes (PVCs) that mediate virulence in grass grubs (16, 35), and (iii) antifeeding prophages (Afp) that cause cessation of feeding and the death of grass grub larvae (17–21). A fourth CIS from “*Candidatus* Amoebophilus asiaticus” (*Bacteroidetes* phylum) (13) promotes intracellular survival in amoeba and defines the subtype 4 T6SS group. Examples of this subtype 4 T6SS group have been functionally confirmed in “*Ca.* Amoebophilus asiaticus” (13) and identified *in silico* in a few other *Bacteroidetes* genomes (22, 23). Recently, a genome-wide identification of bacterial extracellular contractile injection systems predicted more than 50 *Bacteroidetes* eCIS gene clusters, including in *Bacteroidales* from the human gut (24).

In this study, we extend the previously known diversity of *Bacteroidales* species that encode distinct CIS within their genomes, which we term *Bacteroidales* injection systems (BIS). BIS are related to eCIS and T6SS that mediate tubeworm, insect, and amoeba interactions (MACs, PVCs, Afp, subtype 4 T6SS). Here, we show that BIS genes are present within the gut microbiomes of over 99% of healthy human adult individuals from Western countries (Europe and the United States) and are expressed *in vivo*. We further find that individuals suffering from inflammatory bowel disease (IBD) possess fewer BIS gene counts in their gut microbiomes than are in the gut microbiomes of healthy individuals. Our results reveal that genes encoding a putative contractile injection system are carried within the microbiomes of nearly all healthy adults from Western countries and may be correlated with host health.

## RESULTS

### *Bacteroidales* bacteria from the human gut possess genes encoding a putative and distinct contractile injection system.

Using PSI-BLAST to compare previously identified eCIS and subtype 4 T6SS proteins to proteins in the nonredundant (nr) protein sequence database, we identified CIS structural proteins (baseplate, sheath, and tube) that matched proteins from various human *Bacteroidales* isolates, including a bacterial isolate from the human gut, Bacteroides cellulosilyticus WH2 (25), Bacteroides fragilis BE1, and Parabacteroides distasonis D25 (see Table S1 in the supplemental material). To determine the relatedness of these distinct CIS with all known CIS subtypes, we performed phylogenetic analyses of CIS proteins that are key structural components of known CIS: the CIS sheath and tube. Multiple methods of phylogenic analyses (maximum likelihood, neighbor joining, maximum parsimony, unweighted pair group method using average linkages [UPGMA], and minimum evolution) showed that *Bacteroidales* sheath and tube proteins consistently formed a monophyletic group with other eCIS and subtype 4 T6SS sheath and tube proteins (Fig. 1B; Fig. S1; Table S2). Moreover, the BIS sheath and tube were clearly distinct from previously characterized T6SS of subtypes 1, 2, and 3, including *Bacteroides* subtype 3 T6SS (Fig. 1B; Fig. S1) (9, 10). Based on these data and results below, we name these distinct CIS *Bacteroidales* injection systems (BIS).

10.1128/mSystems.00648-20.1 FIG S1 Unrooted phylogeny of CIS tube protein sequences. A BIS group with a known T6SS of subtype 4 and CIS (orange) are distinct from organisms with a known T6SS of subtype 1 and subtype 2 present in human pathogens and of a known subtype 3, characterized to mediate bacterium-bacterium interactions. Download FIG S1, EPS file, 1.9 MB. Copyright © 2020 Rojas et al. 2020 Rojas et al. This content is distributed under the terms of the Creative Commons Attribution 4.0 International license.

10.1128/mSystems.00648-20.5 TABLE S2 Distinctive structural proteins (tube and sheath) representing diverse contractile injection systems. Download Table S2, DOCX file, 0.02 MB. Copyright © 2020 Rojas et al. 2020 Rojas et al. This content is distributed under the terms of the Creative Commons Attribution 4.0 International license.

### Genes encoding BIS are found in a conserved cluster that forms three different genetic arrangements.

**FIG 2:**
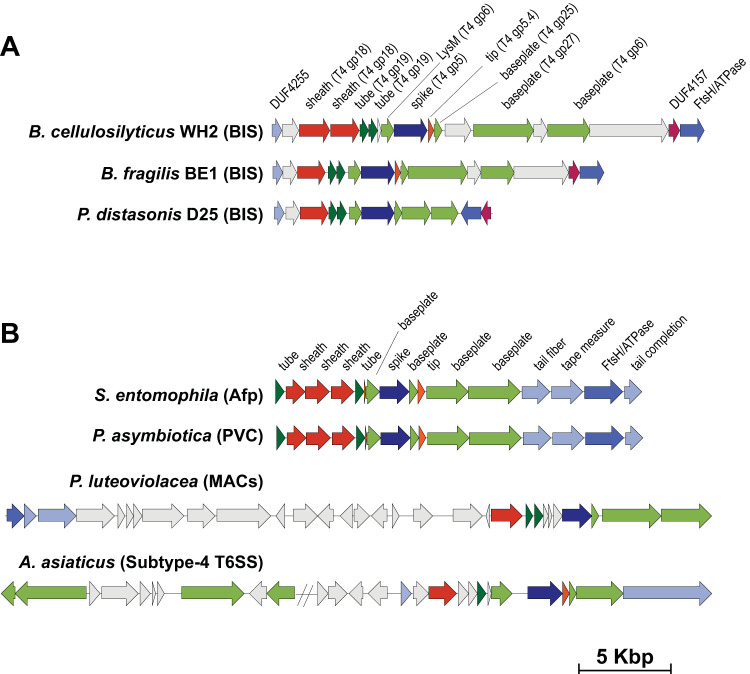
BIS gene clusters are found in three genetic architectures. (A and B) Synteny plot of BIS gene clusters in *Bacteroides* and *Parabacteroides* species (A) compared to those of *P. luteoviolacea* MACs, S. entomophila Afp, *Photorhabdus* PVCs, and “*Ca.* Amoebophilus asiaticus” subtype 4 T6SS (B). Representative CIS gene cluster architectures are shown, with genes color coded according to function. Genes with no significant sequence similarity at the amino acid level to any characterized proteins are light gray. Sequence coordinates of all gene clusters are provided in Table S3.

To identify *Bacteroidales* species that possess a bona fide BIS gene cluster, we performed a comprehensive search of 759 sequenced *Bacteroides* and *Parabacteroides* genomes from the RefSeq database. Our sequence-profile search revealed 66 genomes from *Bacteroides* and *Parabacteroides* species that harbor complete BIS gene clusters (Table S3) in three conserved gene arrangements (Fig. 2A). The first architecture is exemplified by B. cellulosilyticus WH2, which harbors two sheath proteins, two tube proteins, and a protein of unknown function intervening between putative genes encoding the baseplate (*gp25*, *gp27*, and *gp6*). The second architecture is exemplified by B. fragilis BE1. This architecture has a single sheath protein and lacks the hypothetical proteins observed in architecture 1 between gp25 and gp27 and between Tube2 and LysM. The third architecture defined by P. distasonis D25 is the most compact and lacks four hypothetical proteins found in architectures 1 and 2. Additionally, gp27 and gp6 proteins are shorter, and the FtsH/ATPase and DUF4157 genes are inverted. Importantly, all three genetic architectures have genes with significant sequence similarity (E value < 0.001) to MAC, Afp, and PVC genes (Table S1), shown previously to produce a functional CIS, including baseplate proteins (gp25, gp27, and gp6), the sheath, the tube, and FtsH/ATPase (Fig. 2B). These genes were also independently identified in the dbeCIS (database of extracellular contractile injection systems) (24).

10.1128/mSystems.00648-20.6 TABLE S3 Sequence coordinates of genes within the BIS clusters that form three different genetic arrangements. Download Table S3, DOCX file, 0.02 MB. Copyright © 2020 Rojas et al. 2020 Rojas et al. This content is distributed under the terms of the Creative Commons Attribution 4.0 International license.

### BIS genes are present in human gut, mouth, and nose microbiomes.

**FIG 3:**
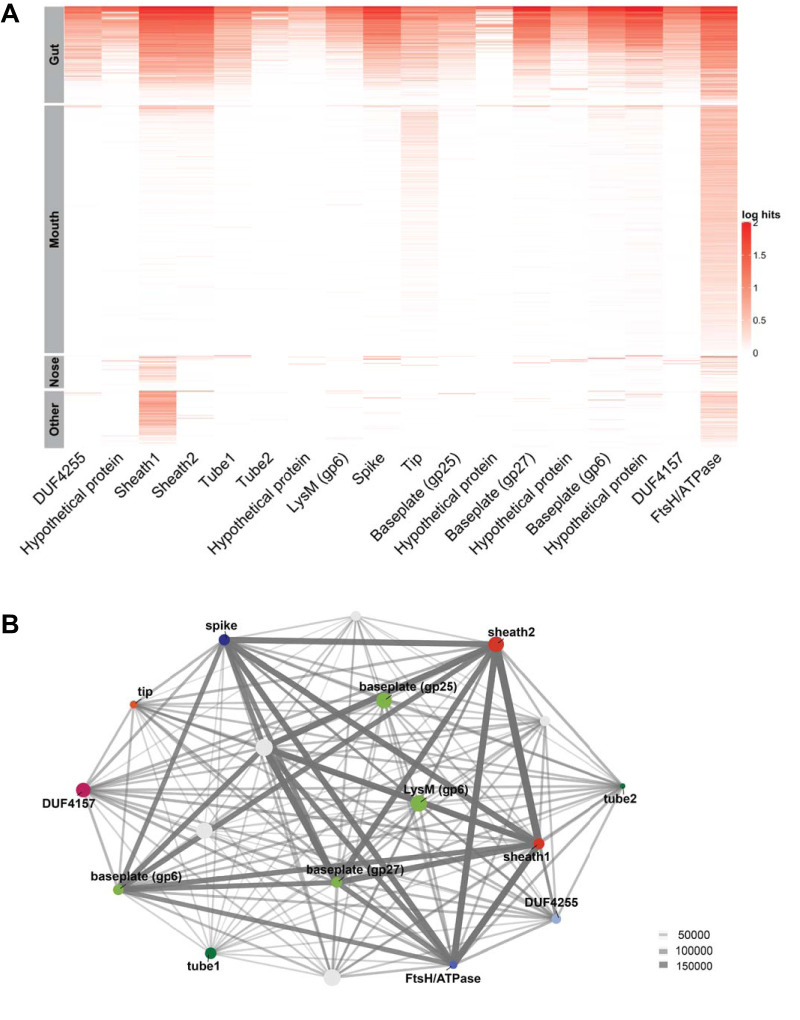
BIS genes are abundant in human gut and mouth microbiomes and present in other human microbiomes. (A) Coverage plot of BIS genes (log_10_ of 1,000,000 · hits/reads) in 8,320 microbiomes associated with mucosal tissue, i.e., gut, mouth, nose, and other (includes vaginal and skin) tissues from 232 healthy humans. (B) Ten BIS genes are often found together in human metagenomes (cooccurrence network). Node size represents the number of hits for each protein across all runs. Line weight represents the number of times that any two proteins occurred together within a data set.

To determine the prevalence and distribution of BIS genes in human microbiomes, we searched shotgun DNA sequencing data from 11,219 microbiomes from the Human Microbiome Project (HMP) database, taken from several locations on the human body of 232 individuals (8, 26). We sampled these metagenomes for the presence of 18 predicted BIS proteins (Table S4). Across all HMP metagenomes, 8,320 (74%) showed hits to at least 1 of the 18 BIS proteins. Hits were distributed across metagenomes from various mucosal tissues and were more abundant in the gut and in the mouth, where *Bacteroidales* are commonly found (8). The data set included stool (1,851 hits, 99.6% of total stool metagenomes), oral (4,739 hits, 79.2% of total oral metagenomes), nasal (630 hits, 41.8% of total nasal metagenomes), and vaginal (232 hits, 27.7% of total vaginal metagenomes) samples (Fig. 3A).

10.1128/mSystems.00648-20.7 TABLE S4 Available annotations for the BIS gene cluster. Download Table S4, DOCX file, 0.01 MB. Copyright © 2020 Rojas et al. 2020 Rojas et al. This content is distributed under the terms of the Creative Commons Attribution 4.0 International license.

To determine how often any of the 18 genes cooccurred within the same metagenome sample, we constructed a cooccurrence network (Fig. 3B). Ten genes appeared together at high frequencies, including those for Sheath1, Sheath2, FtsH/ATPase, Baseplate (gp25, gp27, and gp6), LysM, Spike, and two hypothetical proteins (Fig. 3B). The gene with the highest hit abundance encodes an ATPase homologous to Escherichia coli FtsH, known to be involved in cleavage of the lambda prophage repressor, followed by a hypothetical protein and Sheath1. The remaining genes, including Tube1, Tube2, Tip, DUF4255 domain-containing protein, DUF4157 domain-containing protein, and three hypothetical proteins, were detected together less often within the microbiome samples.

### BIS genes are expressed *in vivo* in the guts of humanized mice and *in vitro* when cultured with various polysaccharides.

**FIG 4:**
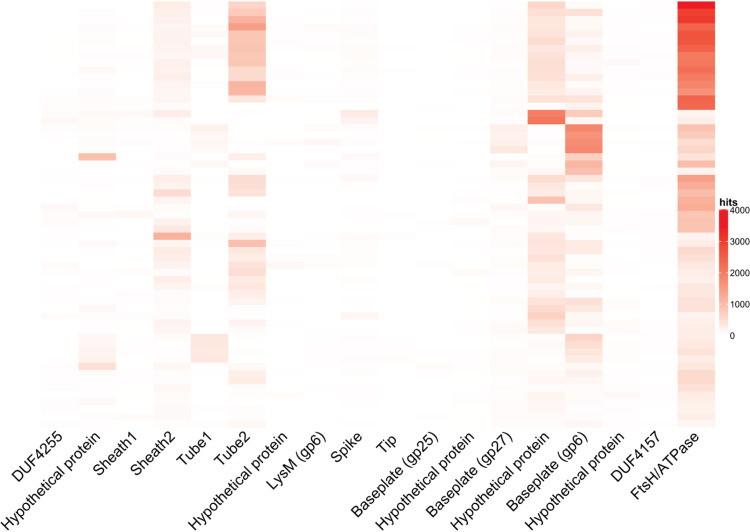
BIS genes are expressed *in vivo* in humanized mice. Coverage plot of BIS genes (normalized by number of reads and protein nucleotide size) from 59 stool metatranscriptomes of humanized mouse microbiomes.

To determine whether BIS genes are transcribed inside the gut or under laboratory growth conditions, we searched for the 18 major BIS proteins in publicly available RNA sequencing data from *in vivo* metatranscriptomes of humanized mice (27) (gnotobiotic mice colonized with human microbiome bacteria) and *in vitro B. cellulosilyticus* WH2 pure cultures (25). We inspected 59 metatranscriptomes from a previously published *in vivo* study (27) for the presence of the 18 major BIS proteins, where gnotobiotic mice were inoculated with human gut microbiome cultures. In 48 out of 59 metatranscriptomes (81.4%), we found expression of at least 15 BIS proteins (Fig. 4). Similarly, when *B. cellulosilyticus* WH2 was cultured in minimum medium (MM) supplemented with 31 different simple and complex sugars (25), all 18 genes were expressed at least once in at least two of the three replicate cultures (Fig. S2). The highest expression was seen under growth in *N*-acetyl-d-galactosamine (GalNAc) and *N*-acetyl-glucosamine (GlcNAc), amino sugars that are common components of the bacterial peptidoglycan, in high abundance in the human colon, and implicated in many metabolic diseases (28, 29). Our analyses of metatranscriptomes show that BIS genes are transcribed by *Bacteroidales* bacteria under laboratory growth conditions and within humanized mouse microbiomes *in vivo*.

10.1128/mSystems.00648-20.2 FIG S2 BIS genes are expressed during *in vitro* culture of *B. cellulosilyticus* WH2. Relative abundances of RNA hits to 18 major genes of the BIS in a *B. cellulosilyticus* WH2 culture in MM supplemented with 31 different simple and complex carbohydrates. Download FIG S2, EPS file, 3.1 MB. Copyright © 2020 Rojas et al. 2020 Rojas et al. This content is distributed under the terms of the Creative Commons Attribution 4.0 International license.

### BIS genes are present in the microbiomes of nearly all adult individuals.

**FIG 5:**
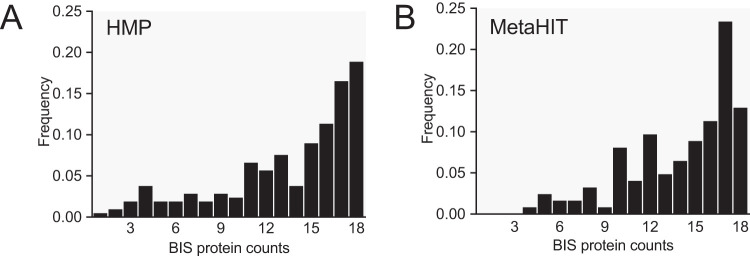
BIS genes are present in the microbiomes of a majority (99%) of adult individuals from the United States and Europe. Frequencies of 18 BIS proteins from fecal samples of 338 individuals are shown. (A) HMP (*n* = 214) from healthy North American individuals (8); (B) MetaHIT (*n* = 124) from a study of European individuals (30). Protein hits are normalized by individual donor.

To determine the prevalence of BIS genes within the microbiomes of human populations, we analyzed 2,123 fecal metagenomes from 339 individuals; 124 individuals from Europe from the MetaHIT (Metagenomics of the Human Intestinal Tract) study (30), and 214 individuals from North America from the HMP study (8). In both the MetaHIT and HMP studies, the cohort of individuals was sequenced more than once; to account for this, the BIS prevalence analysis was normalized by individual donor. We found that all individuals possessed at least 1 of the 18 BIS genes within their gut microbiome (Fig. 5). Most individuals carried at least 9 BIS proteins (83.0% HMP, 90.3% MetaHIT). A lower number possessed all 18 BIS proteins (8.96% HMP, 6.45% MetaHIT).

### BIS genes are more abundant in the gut microbiomes of healthy individuals than in individuals suffering from IBD.

**FIG 6:**
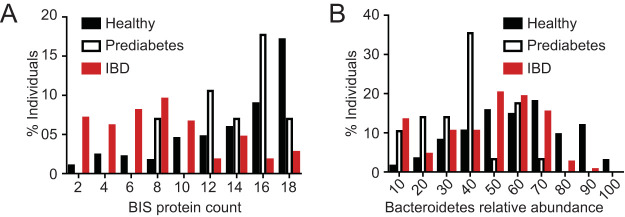
BIS genes are present in higher abundance in healthy individuals than in individuals with IBD. (A) Percentages of individuals possessing a given number of BIS proteins from 214 healthy, 103 IBD, and 28 prediabetes fecal microbiome samples; (B) percentages of individuals possessing a given relative abundance (percentage) of *Bacteroidetes* within their gut microbiomes.

Individuals suffering from inflammatory bowel disease (IBD) and prediabetes have been shown to possess an altered gut microbiome composition (31, 32), yet it is unknown whether specific microbial factors contribute to healthy or diseased outcomes. We asked whether individuals with IBD or prediabetes differ from healthy individuals in their counts of BIS genes within their microbiomes. To this end, we analyzed 4,918 fecal metagenomes from 345 individuals comprising the HMP and the Integrative Human Microbiome Project (iHMP) data set (33): 214 healthy individuals, 103 with IBD (Crohn’s disease or ulcerative colitis), and 28 with prediabetes. For those individuals with more than one metagenome sequence, BIS gene hits were averaged by donor to account for the existence of more than one sequenced metagenome per individual. While prediabetes and healthy individuals possessed comparable counts of BIS genes, we found that a higher percentage of healthy individuals harbored significantly more BIS genes than individuals with IBD (Fig. 6A; Table 1; Fig. S3).

**TABLE 1:** Statistical analyses of BIS protein counts and *Bacteroidetes* abundance confidence intervals for the difference in frequency medians between healthy, prediabetes, and IBD groups by a percentile nonparametric bootstrap method^*a*^

Groups	BIS protein count (95% CI)	*Bacteroidetes* abundance (95% CI)
Healthy vs IBD	0.444* (0.349, 0.505)	0.126* (0.058, 0.184)
Healthy vs prediabetes	0.040 (–0.044, 0.163)	0.218* (0.153, 0.302)
Prediabetes vs IBD	0.404* (0.259, 0.484)	–0.092 (–0.186, –0.033)

a The estimated difference in medians and the corresponding 95th-percentile confidence intervals (95% CI) are reported. Confidence intervals that do not cover zero have significantly different medians, denoted with an asterisk. See Table S5 in the supplemental material for asymptotic Wilcoxon rank sum test results.

10.1128/mSystems.00648-20.3 FIG S3 BIS protein abundance per individual in microbiomes of healthy, prediabetes, and IBD groups. Mean numbers of hits for each protein are normalized by gene size (nucleotides). Error bars indicate standard deviations from results for 214 healthy, 103 IBD, and 28 prediabetes individuals. Download FIG S3, EPS file, 1.2 MB. Copyright © 2020 Rojas et al. 2020 Rojas et al. This content is distributed under the terms of the Creative Commons Attribution 4.0 International license.

10.1128/mSystems.00648-20.8 TABLE S5 Asymptotic Wilcoxon rank sum test results of the analyses of BIS protein counts and *Bacteroidetes* abundances. Download Table S5, DOCX file, 0.01 MB. Copyright © 2020 Rojas et al. 2020 Rojas et al. This content is distributed under the terms of the Creative Commons Attribution 4.0 International license.

We next reasoned that differences in total *Bacteroidetes* abundances between healthy and IBD individuals could account for the differences in BIS counts that we observed. We therefore quantified the abundances of *Bacteroidetes* between healthy, prediabetes, and IBD groups. Our analyses showed that the relative abundances of *Bacteroidetes* per individual were similar between the three groups (Fig. 6B), yet there were statistically different frequency medians per individual between the healthy, prediabetes, and IBD groups (Table 1). Our results show that BIS gene counts are more abundant in healthy individuals than in those with IBD and that these BIS gene counts cannot be explained by the difference in *Bacteroidetes* frequencies between healthy and IBD groups.

## DISCUSSION

Here, we show that a gene cluster encoding a putative contractile injection system, called BIS, is present in the gut microbiomes of nearly all healthy adult individuals from Western countries. We find that BIS genes are present in human microbiomes throughout mucosal tissues (oral, nasal, vaginal, ocular) and enriched in metagenomes from gut samples. Type 6 secretion systems have gained recent recognition as secretion structures that promote disease in several prominent human pathogens, like Pseudomonas aeruginosa and Vibrio cholerae. However, our discovery that a distinct *Bacteroidales*-borne CIS gene cluster is present in a majority of human gut microbiomes stems from studies of symbiotic interactions between environmental bacteria and diverse eukaryotic hosts, like tubeworms, insects, and amoeba (5, 13, 14, 34, 35). The close relatedness of BIS with other structures promoting microbe-eukaryote interactions (MACs, Afp, PVCs, and subtype 4 T6SS) suggests that BIS may mediate interactions between *Bacteroidales* and their human host or bacterial species within the human microbiome. Importantly, our results warrant future investigations into the potential functions of BIS genes in the gut microbiome, which will require significant experimental validation.

Although contractile injection systems (eCIS and T6SS subtypes 1 to 4) share certain components, CIS subtypes are distinguished based on sequence similarity of gene or protein homologs and the presence or absence of specific CIS components. Specifically, the protein sequences of BIS tube, baseplate, and sheath genes possess significant similarity to the homologous proteins of other eCIS, based on E value (see Table S1 in the supplemental material). We used metagenomic and metatranscriptomic analyses with thresholds (E value < 0.001) that excluded homologous genes from other secretion systems (E value > 0.01). Further, a distinguishing feature of gene clusters related to MACs, Afps, PVCs, and BIS is the presence of a baseplate (*gp27*) gene. This *gp27* gene is not present in canonical T6SS subtypes 1 to 3 and is likely to correspond to BIS gene clusters when identified within our metagenomic analyses. Independent methods have been used previously to find, describe, and characterize 631 eCIS-like loci from the 11,699 publicly available complete bacterial genomes, including BIS (24). We acknowledge that these analyses come with potential limitations and that experimental validations are required to support the findings described here.

BIS may not have been extensively described before this work because they likely evolved independently from previously described CIS, such as subtype 3 T6SS (12, 13), and possess significantly divergent sequence homologies (Fig. 1B). Like other described CIS, BIS gene clusters harbor genes encoding the syringe-like structural components and may encode effectors that elicit specific cellular responses from target cells. For example, the closely related injection system called MACs possesses two different effectors; one effector protein promotes the metamorphic development of a tubeworm (15), and a second toxic effector kills insect and mammalian cell lines (36).

We currently do not yet know the conditions that promote BIS production within healthy or diseased human individuals. However, we show here that BIS genes are expressed *in vivo* during colonization of humanized mice (27) and under laboratory conditions with various carbon sources (25). We observed a heterogenous expression pattern of BIS genes in humanized mouse transcriptomes and *in vitro*, which may be due to limitations in sequencing depth and/or differential expression of BIS structural and effector proteins that compose a multisubunit complex.

Our analyses show that BIS genes are more prevalent in individuals with healthy gastrointestinal tracts than in those suffering from IBD (Crohn’s disease and ulcerative colitis). Several studies have demonstrated that dysbiosis in the human gut is correlated with microbiome immaturity, type 2 diabetes, and diseases like obesity and inflammatory bowel disease (IBD) (31, 37–41). IBD is a chronic inflammation of the gastrointestinal tract that encompasses two diseases, Crohn’s disease and ulcerative colitis, both characterized by decreased microbial diversity, lower microbiome composition stability, and an increase in *Enterobacteria* (42, 43). Studies have shown that although dysbiosis and the metabolic profiles of the gut microbiome influence the disease, microbial functions have a greater contribution to the disease (42). Our work warrants future investigations into whether BIS play a role in the promotion or maintenance of a healthy gastrointestinal tract.

If BIS do interact with human cells, they may promote or enhance symbiotic interactions with human gut commensals, such as Bacteroides cellulosilyticus (44). Injection systems closely related to BIS are described to mediate both beneficial and infectious microbe-host relationships. For example, MACs mediate metamorphosis of marine tubeworms (5, 15), and a subtype 4 T6SS (13) mediates membrane interaction between “*Ca*. Amoebophilus asiaticus” and its amoeboid host. In contrast, Afp and PVCs inject toxic effectors into insects (16, 20). Our findings evidence the presence of the BIS gene cluster in a majority of human gut microbiomes; however, the potential function of the BIS genes needs to be investigated. The hypothesis that the BIS cluster may mediate microbe-human interactions arises from previous studies that describe, and characterize, similar gene clusters (5, 13, 16).

Many correlations between *Bacteroidales* abundances in the human gut and host health are currently unexplained. Little is known about the cross talk mechanisms between the microbiome and the human host and the functional role of *Bacteroidales* in microbial dynamics in the human gut. Future research into the conditions that promote the production of BIS and its potential protein effectors may yield new insight into how *Bacteroidales* prevalence correlates with host health. In addition to having an effect on host health, functional BIS may provide the tantalizing potential as biotechnology platforms that may be manipulated to inject engineered proteins of interest into other microbiome bacteria or directly into human cells.

## MATERIALS AND METHODS

### Phylogenetic analyses of CIS sheath and tube proteins.

Whole genomes and assembled contigs illustrating a diversity of representative phage-like clusters (see Table S2 in the supplemental material) were downloaded from the NCBI data bank to construct a database using BLAST+(2.6.0). The *B. cellulosilyticus* WH2 Sheath1 (NCBI Protein database accession no. WP_029427210.1) and Tube1 (WP_118435218) protein sequences were downloaded, and a tBLASTn search was performed against the genome database. The recovered nucleotide sequences were then translated using EMBOSS Transeq (EMBL-EBI, https://www.ebi.ac.uk/Tools/st/emboss_transeq/) to generate a list of protein sequences. While the sheath and tube proteins are functionally homologous, their genomic diversity required other protein queries against the custom genome database to capture protein homologs from more divergent secretion systems (sheaths, NCBI Protein database accession no. WP_012025251.1, YP_009591452.1, WP_001882966.1; tubes, WP_012473180.1, YP_009591453.1, WP_015969329.1, WP_003022149.1, WP_001142947.1). To capture these highly divergent protein homologs, we used T6SS-Hcp and VipA/B and phage gp18 and gp19 as reference proteins. The amino acid sequences were aligned using the online version of MAFFT (v7) with the iterative refinement alignment method e-ins-i for the Sheath1 phylogeny and fft-ins-I for the Tube1 phylogeny. The aligned fasta file was converted into a Phylip file using Seaview (45). PhyML was performed through the ATCG Bioinformatics Web server and utilized the Smart Model Selection (SMS) feature and the maximum likelihood method (46, 47). The model WAG+G+I+F was used for the Sheath1, and rtREV+G+I+F was used for the Tube1 phylogeny. Different alignment algorithms were used based on the conservation of the protein sequences. The smart model selection feature from the ATGC Web server calculated the best phylogenetic substitution model based on the alignment. Bootstrap values of 1,000 resamples (instead of only 100) were calculated to ensure tree robustness. The maximum likelihood tree topology was confirmed using other methods, including neighbor joining, maximum parsimony, minimum evolution, and UPGMA. Trees were manipulated and viewed in iTOL (48).

### BIS gene cluster synteny analyses.

To identify CIS gene clusters in *Bacteroidetes*, we used a modified protocol to identify T6SS (12). Briefly, the assemblies for 759 *Bacteroides* and *Parabacteroides* genomes included in the RefSeq database (release 92, 26553804) were downloaded. Proteins from each assembly were searched with HHMER v3.2.1 (http://hmmer.org/) for a match above the sequence gathering threshold (bit score > 31.4, E value < 1 × 10^−9^) of the Pfam HMM profile “phage_sheath_1” (PF04984) (49). For each match, up to 20 proteins were extracted from either side. All proteins from the resulting set (phage sheath ± 20 proteins) were sorted by length and clustered at 50% amino acid identity using UClust v1.2.22q (50). Clusters containing ≥4 members were analyzed further. Cluster representatives were annotated using protein profile searches against three databases: the Pfam-A database using HMMER3 (using family-specific gathering thresholds) (49), the NCBI Conserved Domain Database using RPS-BLAST (E value < 0.01) (51–53), and the Uniprot30 database (accessed February 2019, available from http://wwwuser.gwdg.de/~compbiol/data/hhsuite/databases/hhsuite_dbs/) using HHblits (54, 55). Multiple sequence alignments were automatically generated from three iterations of the HHblits search and used for profile-profile comparisons against the PDB70 database (HHpred probability > 90, accessed February 2019, available from http://wwwuser.gwdg.de/~compbiol/data/hhsuite/databases/hhsuite_dbs/). Significant hits to cluster representatives were used to assign an annotation to all proteins contained within the parent cluster. Manual inspection of *Bacteroides* and *Parabacteroides* loci enabled consistent trimming of each genetic architecture; specifically, the genes intervening between DUF4255 and FtsH/ATPase were retained.

### Metagenomic mining analyses.

To find the prevalence of the BIS genes within the Human Microbiome Project and the Integrative HMP, using NCBI’s fastq-dump API, we downloaded 11,219 and 3,059 metagenomes, respectively. The metagenomes were parsed where left-right tags were clipped, technical reads (adapters, primers, barcodes, etc.) were dropped, low-quality reads were dropped, and paired reads were treated as two distinct reads. A subject database was created from the amino acid sequences of the 18 BIS genes. Then the fastq files were piped through seqtk (56) to convert them to fasta format, which was then piped to DIAMOND via stdin. Then DIAMOND aligned the six-frame translation of the input reads against the subject database, with all default parameters and an E value cutoff of 0.001. For each metagenome, the number of nonmutually exclusive hits to each CIS gene were then summed providing a hit “count score.” From the hit counts, a heatmap was created by taking the number of hits of each gene per metagenome and dividing that number by the total number of reads and multiplying the result by 1 million, which was then log_10_(*x* + 1) transformed. To estimate the cooccurrence between pairs of genes, the hit count scores from the previous calculation were taken, and for each pair combination, the hit count of the lower of the two genes was added to a running total. The cooccurrence was then visualized on a network graph, where each edge corresponds to the number of times the pair of genes cooccurred in all the metagenomes (57, 58) (R core Team 2017, https://www.R-project.org/; ggraph, https://CRAN.R-project.org/package=ggraph). The prevalence of BIS genes was normalized by human donor to account for the presence of more than one sequenced metagenome per individual. For the MetaHIT and HMP data sets, the average number of BIS proteins per person was calculated based on the metadata provided by the studies.

### Metatranscriptomic mining analyses.

Fastq files from transcriptomes were downloaded from the Sequence Read Archive using the SRA Toolkit (https://www.ncbi.nlm.nih.gov/sra/docs/sradownload/). Low-quality reads were removed using PRINSEQ++ (https://peerj.com/preprints/27553/). Reads were compared to the amino acid sequences of the Bacteroides cellulosilyticus WH2 BIS protein cluster using BLASTx and an E value cutoff of 0.001. The best hit for each read was kept. Hits to each protein were normalized by the number of reads of each transcriptome and the length of each protein using the program Fragment Recruitment Assembly Purification (https://github.com/yinacobian/frap).

### *Bacteroidetes* abundance in healthy, IBD, and prediabetes microbiomes.

To estimate bacterial taxonomy abundance, MetaPhlAn version 2.6.0 was downloaded, along with the corresponding version 20 database, and run on each of the metagenomes from the Human Microbiome Project and the Integrative Human Microbiome Project (59)

### Statistical analysis for comparison of BIS in healthy, IBD, and prediabetes groups.

To test the difference between the medians of two groups (healthy versus IBD, prediabetes versus IBD, and healthy versus prediabetes), a confidence interval (CI) for the difference in medians was constructed by the percentile nonparametric bootstrap method for the difference in medians, using 10,000 bootstrap replicates for each group. Statistical analysis showed no difference between Crohn’s disease and ulcerative colitis for either BIS protein count (Crohn’s disease versus colitis, −0.02519917 [–0.1524929, 0.1018519]) or *Bacteroidetes* abundance (Crohn’s disease versus colitis, 0.01385387 [–0.1265633, 0.1489824]). These results were further validated with asymptotic Wilcoxon rank sum tests (Table S5).

### Availability of data.

The data sets supporting the conclusions of this article are available in the Human Microbiome Project Data Portal (https://portal.hmpdacc.org/); the additional metagenomes and metatranscriptomes analyzed in this study, corresponding to those of previous studies (25, 27, 30), are publicly available in the NCBI SRA database, and all accession numbers and protein IDs are listed in the supplemental material (Tables S1 to S4). Phylogenetic and synteny analyses were performed with Web server programs cited herein. Scripts used for metagenomic and metatranscriptomic data analyses are available in GitHub (https://github.com/yinacobian/MR-blastx).
