# Supplementary material for: A Distinct Contractile Injection System Gene Cluster Found in a Majority of Healthy Adult Human Microbiomes
Source: mSystems. 2020 Jul 28;5(4):e00648-20. doi: 10.1128/mSystems.00648-20 (PMC7394362; doi:10.1128/mSystems.00648-20)
Supplement: TABLE S1 [file mSystems.00648-20-st001.docx]

| **Organism** | **Protein** | **Locus tag (query)** | **e-value** |
| --- | --- | --- | --- |
| ***B. cellulosilyticus* WH2 BASEPLATE locus tag (subj.): WP_029427202.1** | | | |
| *B. fragilis* BIS | hyp. prot. | CUA19277.1 | 3.00E-90 |
| *A. asiaticus* T6SS^iv^ | baseplate | WP_012472726.1 | 2.00E-67 |
| *C. hertigii* T6SS | baseplate | WP_014934193.1 | 2.00E-57 |
| *Parabacteroides sp.* BIS | baseplate | WP_009276509.1 | 1.00E-42 |
| *Flavobacterium johnsoniae* eCIS | baseplate | ABQ06175 | 1.00E-42 |
| *P. luteo* MACs | MacB | WP_029427202.1 | 5.00E-32 |
| *S. entomophila* Afps | baseplate | WP_010895813.1 | 7.00E-18 |
| *P. asymbiotica* PVCs | baseplate | WP_015834374.1 | 3.00E-16 |
| *V. cholerae* T6SS^i^ | baseplate | NP_232421.1 | 0.61 |
| ***B. cellulosilyticus* WH2 SHEATH-1 locus tag (subj.): WP_029427210.1** | | | |
| *Parabacteroides sp.* BIS | sheath2 | WP_009276514.1 | 0.00E+00 |
| *B. fragilis* BIS | sheath2 | WP_005803145.1 | 2.00E-109 |
| *P. luteo* MACs | MacS | WP_039609824.1 | 6.00E-94 |
| *A. asiaticus* T6SS^iv^ | sheath | WP_012473177.1 | 5.00E-66 |
| *C. hertigii* T6SS^iv^ | sheath | WP_014934609.1 | 8.00E-65 |
| *Flavobacterium johnsoniae* eCIS | sheath2 | WP_012025137.1 | 2.00E-64 |
| *P. asymbiotica* PVCs | sheath1 | WP_015834924.1 | 2.00E-51 |
| *S. entomophila* Afps | sheath1 | WP_010895805.1 | 9.00E-49 |
| *Pseudomonas aeruginosa* eCIS | sheath1 | WP_003113197.1 | 8.00E-10 |
| *Salmonella enterica subsp. enterica serovar Typhi* eCIS | sheath1 | WP_000046142.1 | 8.00E-06 |
| *Francisella tularensis subsp. tularensis*  T6SS^ii^ | sheath | WP_003023948.1 | 2.10E-01 |
| *Parabacteroides sp.* T6SS^iii^ | sheath1 | WP_008669225.1 | 2.30E-01 |
| *B. fragilis* T6SS^iii^ | sheath1 | WP_053873779.1 | 4.90E-01 |
| *Flavobacterium johnsoniae* T6SS^iii^ | sheath1 | WP_012025251.1 | 4.90E-01 |
| *Pseudomonas aeruginosa* T6SS^i^ | sheath2 | WP_003087596.1 | 8.30E-01 |
| *Salmonella enterica subsp. enterica serovar Typhi* T6SS | sheath2 | WP_000013884.1 | 1.30E+00 |
| *V. cholerae* T6SS^i^ | sheath | WP_001882966.1 | 3.30E+00 |
| ***B. cellulosilyticus* WH2 SHEATH-2 locus tag (subj.): WP_029427209.1** | | | |
| *B. fragilis* BIS | sheath2 | WP_005803145.1 | 0.00E+00 |
| *Parabacteroides sp.* BIS | sheath2 | WP_009276514.1 | 0.00E+00 |
| *C. hertigii* T6SS^iv^ | sheath | WP_014934609.1 | 7.00E-122 |
| *A. asiaticus* T6SS^iv^ | sheath | WP_012473177.1 | 3.00E-114 |
| *Flavobacterium johnsoniae* eCIS | sheath2 | WP_012025137.1 | 6.00E-70 |
| *P. asymbiotica* PVCs | sheath2 | WP_015834924.1 | 3.00E-64 |
| *P. luteo* MACs | MacS | WP_039609824.1 | 1.00E-63 |
| *S. entomophila* Afps | sheath2 | WP_010895805.1 | 1.00E-57 |
| *Pseudomonas aeruginosa* eCIS | sheath1 | WP_003113197.1 | 2.00E-08 |
| *Salmonella enterica subsp. enterica serovar Typhi* eCIS | sheath1 | WP_000046142.1 | 4.40E-02 |
| *V. cholerae* T6SS^i^ | sheath | WP_001882966.1 | 2.50E-01 |
| *B. fragilis* T*6SS^iii^* | sheath1 | WP_053873779.1 | 2.90E-01 |
| *Parabacteroides sp.* T6SS^iii^ | sheath1 | WP_008669225.1 | 3.10E-01 |
| *Flavobacterium johnsoniae* T6SS^iii^ | sheath1 | WP_012025251.1 | 5.20E-01 |
| *Pseudomonas aeruginosa* T6SS^i^ | sheath2 | WP_003087596.1 | 2.20E+00 |
| *Francisella tularensis subsp. tularensis*  T6SS^ii^ | sheath | WP_003023948.1 | 2.70E+00 |
| *Salmonella enterica subsp. enterica serovar Typhi* T6SS | sheath2 | WP_000013884.1 | 6.40E+00 |
| ***B. cellulosilyticus* WH2 TUBE-1 locus tag (subj.): WP_007212392.1** | | | |
| *Parabacteroides sp.* BIS | tube2 | WP_005861441.1 | 3.00E-62 |
| *B. fragilis* BIS | tube1 | WP_005803146.1 | 4.00E-59 |
| *Flavobacterium johnsoniae* eCIS | tube2 | WP_007806487.1 | 6.00E-51 |
| *S. entomophila* Afps | tube | WP_010895803.1 | 3.00E-32 |
| *P. luteo* MACs | MacT1 | WP_039609825.1 | 2.00E-31 |
| *P. asymbiotica* PVCs | tube | WP_015835472.1 | 4.00E-31 |
| *P. luteo* MACs | MacT2 | WP_039609826.1 | 1.00E-09 |
| *A. asiaticus* T6SS^iv^ | tube | WP_012473180.1 | 3.00E-08 |
| *C. hertigii* T6SS^iv^ | tube | WP_014934612.1 | 6.00E-05 |
| *V. cholerae* T6SS^i^ | tube | WP_001142947.1 | 2.20E-01 |
| *B. fragilis* T6SS^iii^ | tube2 | WP_005787106.1 | 4.20E-01 |
| *Salmonella enterica subsp. enterica serovar Typhi* eCIS | tube2 | WP_001207653.1 | 5.70E-01 |
| *Flavobacterium johnsoniae* T6SS^iii^ | tube1 | WP_012025247.1 | 1.00E+00 |
| *Salmonella enterica subsp. enterica serovar Typhi T6SS* | tube1 | WP_000338756.1 | 1.30E+00 |
| *Parabacteroides sp.* T6SS^iii^ | tube1 | WP_005836793.1 | 3.80E+00 |
| *Pseudomonas aeruginosa* eCIS | tube2 | WP_003083317.1 | 7.00E+00 |
| *Francisella tularensis subsp. tularensis* | tube | WP_003022149.1 | NA |
| *Pseudomonas aeruginosa* T6SS^i^ | tube1 | WP_003085175.1 | NA |
| ***B. cellulosilyticus* WH2 TUBE-2 locus tag (subj.): WP_007212393.1** | | | |
| *A. asiaticus* T6SS^iv^ | tube | WP_012473180.1 | 3.00E-09 |
| *S. entomophila* Afps | tube | WP_010895803.1 | 9.00E-09 |
| *P. asymbiotica* PVCs | tube | WP_015835472.1 | 1.00E-08 |
| *Flavobacterium johnsoniae* eCIS | tube2 | WP_007806487.1 | 1.00E-08 |
| *P. luteo* MACs | MacT2 | WP_039609825.1 | 6.00E-08 |
| *Parabacteroides sp.* BIS | tube2 | WP_005861441.1 | 2.00E-07 |
| *B. fragilis* BIS | tube1 | WP_005803146.1 | 2.00E-06 |
| *P. luteo* MACs | MacT1 | WP_039609826.1 | 6.00E-06 |
| *C. hertigii* T6SS^iv^ | tube | WP_014934612.1 | 1.00E-04 |
| *Pseudomonas aeruginosa* eCIS | tube2 | WP_003083317.1 | 3.60E-01 |
| *B. fragilis* T6SS^iii^ | tube2 | WP_005787106.1 | 5.70E-01 |
| *Salmonella enterica subsp. enterica serovar Typhi* Ecis | tube1 | WP_000338756.1 | 6.90E-01 |
| *V. cholerae* T6SS^i^ | tube | WP_001142947.1 | 7.60E-01 |
| *Flavobacterium johnsoniae* T6SS^iii^ | tube1 | WP_012025247.1 | 3.90E+00 |
| *Pseudomonas aeruginosa* T6SS^i^ | tube1 | WP_003085175.1 | 6.00E+00 |
| *Parabacteroides sp.* T6SS^iii^ | tube1 | WP_005836793.1 | 6.10E+00 |
| *Francisella tularensis subsp. tularensis* | tube | WP_003022149.1 | NA |
| *Salmonella enterica subsp. enterica serovar Typhi* T6SS | tube2 | WP_001207653.1 | NA |
