# Supplementary material for: A Distinct Contractile Injection System Gene Cluster Found in a Majority of Healthy Adult Human Microbiomes
Source: mSystems. 2020 Jul 28;5(4):e00648-20. doi: 10.1128/mSystems.00648-20 (PMC7394362; doi:10.1128/mSystems.00648-20)
Supplement: TABLE S2 [file mSystems.00648-20-st002.docx]

| **Species** | **Strain** | **Genome Accession** | **Sheath Protein Accession** | **Tube Protein Accession** | **Reference** |
| --- | --- | --- | --- | --- | --- |
| Candidatus *Amoebophilus asiaticus* | 5a2 | CP001102.1 | WP_012473177.1 | WP_012473180.1 | (Böck et al., 2017) |
| *Bacteroides cellulosilyticus* | WH2 | CP012801.1 | WP_029427210.1 | WP_007212392.1 | (McNulty et al., 2013) |
| *Bacteroides fragilis* | BFBE1.1 (BE1) | LN877293.1 | WP_005803145.1, WP_053873779.1 | WP_005803146.1 | This study (synteny analysis) |
| *Cardinium hertigii* | cEper1 | NC_018605.1 | WP_014934609.1 | WP_014934612.1 | (Böck et al., 2017) |
| Enterobacteria phage P2 |  | NC_041848.1 | YP_009591452.1 | YP_009591453.1 | Reference phage |
| Enterobacteria phage T4 |  | NC_000866.4 | NP_049780.1 | WP_015969329.1 | Reference phage |
| *Flavobacterium johnsoniae* | UW101 | NC_009441.1 | WP_012025137.1, WP_012025251.1 | WP_012025138.1 | (Böck et al., 2017) |
| *Francisella tularensis subsp. tularensis* | SCHU S4 | AJ749949.2 | WP_003023948.1 | WP_003022149.1 | (Böck et al., 2017) |
| *Parabacteroides sp.* | D25 | NZ_JH976500.1 | WP_009276514.1, WP_008669225.1 | WP_005861441.1 | This study (synteny analysis) |
| *Pseudoalteromonas luteoviolacea* | HI1 | KF724687.1 | WP_039609824.1 | WP_039609825.1, WP_039609826.1 | (Böck et al., 2017) |
| *Pseudomonas aeruginosa* | PAO1 | NC_002516.2 | WP_003113197.1, WP_003087596.1 | WP_003083317.1, WP_003085175.1 | (Böck et al., 2017) |
| *Salmonella enterica subsp. enterica* serovar Typhi | CT18 | NC_003198.1 | WP_000046142.1, WP_000013884.1 | WP_000338756.1, WP_001207653.1 | (Sana et al., 2016) |
| *Serratia entomophila* (pADAP) | A1MO2 | NC_002523.4 | WP_010895805.1 | WP_010895803.1 | (Hurst et al., 2004) |
| *Vibrio cholerae* O1 biovar El Tor | N16961 | NC_002506.1 | WP_001882966.1 | WP_001142947.1 | (Ishikawa et al., 2012) |
| *Photorhabdus asymbiotica* | ATCC43949 | NC_012962.1 | WP_015835470.1 | WP_015835472.1 | (Vlisidou et al., 2019) |
