# Supplementary material for: A Distinct Contractile Injection System Gene Cluster Found in a Majority of Healthy Adult Human Microbiomes
Source: mSystems. 2020 Jul 28;5(4):e00648-20. doi: 10.1128/mSystems.00648-20 (PMC7394362; doi:10.1128/mSystems.00648-20)
Supplement: TABLE S3 [file mSystems.00648-20-st003.docx]

| **Accession** | **Species** | **Strain** | **start** | **stop** | **strand** | **arch.** |
| --- | --- | --- | --- | --- | --- | --- |
| **NZ_ATFI01000004.1** | ***Bacteroides cellulosilyticus WH2*** | **WH2** | **271076** | **294193** | **-** | **1** |
| NZ_QRXS01000004.1 | *Bacteroides cellulosilyticus* | AF17-25 | 276218 | 298574 | - | 1 |
| NZ_QRVJ01000001.1 | *Bacteroides cellulosilyticus* | AF22-3AC | 604020 | 580904 | + | 1 |
| NZ_QRSV01000007.1 | *Bacteroides cellulosilyticus* | AF29-17 | 269200 | 292317 | - | 1 |
| NZ_LRGD01000010.1 | *Bacteroides cellulosilyticus* | CL09T06C25 | 44848 | 67991 | - | 1 |
| NZ_JH724085.1 | *Bacteroides cellulosilyticus CL02T12C19* | CL02T12C19 | 94259 | 117409 | + | 1 |
| NZ_EQ973491.1 | *Bacteroides cellulosilyticus DSM 14838* | DSM 14838 | 290576 | 313959 | + | 1 |
| NZ_KQ968695.1 | *Bacteroides intestinalis* | KLE1704 | 249385 | 272510 | - | 1 |
| NZ_QSUL01000020.1 | *Bacteroides oleiciplenus* | OM05-15BH | 29099 | 52460 | + | 1 |
| NZ_JH992940.1 | *Bacteroides oleiciplenus YIT 12058* | YIT 12058 | 1533689 | 1555212 | - | 1 |
| NZ_JAGH01000002.1 | *Bacteroides sp. 14(A)* | 14(A) | 2478682 | 2501030 | + | 1 |
| NZ_RAZN01000003.1 | *Parabacteroides goldsteinii* | 0.1X-D42-15 | 28300 | 49757 | + | 1 |
| NZ_JH976474.1 | *Parabacteroides goldsteinii CL02T12C30* | CL02T12C30 | 486316 | 507720 | + | 1 |
| NZ_KE159513.1 | *Parabacteroides goldsteinii dnLKV18* | dnLKV18 | 1159956 | 1181413 | - | 1 |
| NZ_QSWF01000006.1 | *Parabacteroides gordonii* | OM02-37 | 268051 | 289934 | - | 1 |
| NZ_KE386763.1 | *Parabacteroides gordonii DSM 23371* | DSM 23371 | 428020 | 449720 | + | 1 |
| NZ_KQ033920.1 | *Parabacteroides gordonii MS-1* | MS-1 | 770218 | 791918 | - | 1 |
| NZ_QUHH01000016.1 | *Parabacteroides sp. AF14-59* | AF14-59 | 19676 | 38492 | + | 1 |
| NZ_QTMM01000005.1 | *Parabacteroides sp. AF17-3* | AF17-3 | 296338 | 317796 | - | 1 |
| NZ_QUGR01000001.1 | *Parabacteroides sp. AF18-52* | AF18-52 | 476916 | 495726 | - | 1 |
| NZ_QUDI01000023.1 | *Parabacteroides sp. AF48-14* | AF48-14 | 70961 | 91946 | + | 1 |
| NZ_KB822571.1 | *Parabacteroides sp. ASF519* | ASF519 | 5930811 | 5952268 | - | 1 |
| NZ_KQ033902.1 | *Parabacteroides sp. HGS0025* | HGS0025 | 2808277 | 2829908 | + | 1 |
| NZ_LT669941.1 | *Parabacteroides timonensis* | Marseille-P3236 | 862055 | 883911 | - | 1 |
| **NZ_LN877293.1** | ***Bacteroides fragilis*** | **BE1** | **3202545** | **3220402** | **-** | **2** |
| NZ_QRZO01000002.1 | *Bacteroides fragilis* | AF14-14AC | 123788 | 141668 | - | 2 |
| NZ_QRZH01000002.1 | *Bacteroides fragilis* | AF14-26 | 126212 | 144092 | - | 2 |
| NZ_AKBY01000003.1 | *Bacteroides fragilis CL05T00C42* | CL05T00C42 | 80558 | 98415 | - | 2 |
| NZ_JH724200.1 | *Bacteroides fragilis CL05T12C13* | CL05T12C13 | 80522 | 98379 | - | 2 |
| NZ_JGDN01000068.1 | *Bacteroides fragilis str. 3397 N2* | 3397 N2 | 69183 | 87040 | - | 2 |
| NZ_JGEG01000075.1 | *Bacteroides fragilis str. 3397 N3* | 3397 N3 | 69898 | 87755 | - | 2 |
| NZ_JGDO01000041.1 | *Bacteroides fragilis str. 3397 T14* | 3397 T14 | 2229 | 20086 | - | 2 |
| **NZ_JH976506.1** | ***Parabacteroides sp. D25*** | **D25** | **344282** | **355992** | **+/-** | **3** |
| NZ_GG705151.1 | *Bacteroides sp. 2_1_33B* | 2_1_33B | 811723 | 828493 | +/- | 3 |
| NZ_CYXP01000003.1 | *Parabacteroides distasonis* | 2789STDY5608872 | 797 | 17567 | +/- | 3 |
| NZ_CZAR01000002.1 | *Parabacteroides distasonis* | 2789STDY5834901 | 1098 | 17868 | +/- | 3 |
| NZ_CZBM01000003.1 | *Parabacteroides distasonis* | 2789STDY5834948 | 348507 | 365278 | +/- | 3 |
| NZ_QRXK01000024.1 | *Parabacteroides distasonis* | AF18-10 | 690 | 12400 | +/- | 3 |
| NZ_QRPA01000023.1 | *Parabacteroides distasonis* | AF36-3 | 57479 | 74255 | +/- | 3 |
| NZ_NFJX01000005.1 | *Parabacteroides distasonis* | An199 | 298514 | 315279 | +/- | 3 |
| NZ_NNCA01000001.1 | *Parabacteroides distasonis* | CBA7138 | 2878268 | 2889978 | +/- | 3 |
| NZ_JH976489.1 | *Parabacteroides distasonis CL09T03C24* | CL09T03C24 | 423568 | 440338 | +/- | 3 |
| NZ_JNHK01000089.1 | *Parabacteroides distasonis str. 3776 D15 i* | 3776 D15 i | 3655 | 25273 | +/- | 3 |
| NZ_JNHU01000057.1 | *Parabacteroides distasonis str. 3776 D15 iv* | 3776 D15 iv | 236310 | 253075 | +/- | 3 |
| NZ_JNHL01000054.1 | *Parabacteroides distasonis str. 3776 Po2 i* | 3776 Po2 i | 82522 | 99287 | +/- | 3 |
| NZ_KQ236096.1 | *Parabacteroides sp. 2_1_7* | 2_1_7 | 1621793 | 1633503 | +/- | 3 |
| NZ_QSQY01000029.1 | *Parabacteroides sp. 20_3* | TF09-4 | 28656 | 45426 | +/- | 3 |
| NZ_QSQL01000017.1 | *Parabacteroides sp. 20_3* | TF12-11 | 628 | 17398 | +/- | 3 |
| NZ_QTMJ01000002.1 | *Parabacteroides sp. AF19-14* | AF19-14 | 758 | 17529 | +/- | 3 |
| NZ_QTMG01000015.1 | *Parabacteroides sp. AF21-43* | AF21-43 | 616 | 17386 | +/- | 3 |
| NZ_QTLZ01000003.1 | *Parabacteroides sp. AF27-14* | AF27-14 | 356010 | 372781 | +/- | 3 |
| NZ_QTLP01000005.1 | *Parabacteroides sp. AF39-10AC* | AF39-10AC | 654 | 17424 | +/- | 3 |
| NZ_QTLI01000013.1 | *Parabacteroides sp. AM17-47* | AM17-47 | 642 | 17412 | +/- | 3 |
| NZ_QTLD01000037.1 | *Parabacteroides sp. AM25-14* | AM25-14 | 1411 | 18181 | +/- | 3 |
| NZ_RAYG01000046.1 | *Parabacteroides sp. CH2-D42-20* | CH2-D42-20 | 801 | 17566 | +/- | 3 |
| NZ_KQ236106.1 | *Parabacteroides sp. D26* | D26 | 373154 | 389924 | +/- | 3 |
| NZ_QTMX01000003.1 | *Parabacteroides sp. OF01-14* | OF01-14 | 867 | 12577 | +/- | 3 |
| **NZ_LIDT01000035.1** | ***Bacteroides fragilis*** | **20793-3** | **14633** | **36151** | **-** | **Tn** |
| NZ_PDCT01000010.1 | *Bacteroides fragilis* | CM1`3 | 14711 | 36229 | - | Tn |
| NZ_QTLE01000029.1 | *Bacteroides sp. AM23-18* | AM23-18 | 24670 | 49228 | + | Tn |
| NZ_QRZK01000006.1 | *Bacteroides thetaiotaomicron* | AF14-20 | 31752 | 54562 | + | Tn |
| NZ_QROV01000008.1 | *Bacteroides thetaiotaomicron* | AF37-12 | 31743 | 54552 | + | Tn |
| NZ_QRKS01000015.1 | *Bacteroides thetaiotaomicron* | AM15-10 | 56434 | 79243 | + | Tn |
| NZ_QSLC01000002.1 | *Bacteroides thetaiotaomicron* | AM26-17LB | 486821 | 509631 | - | Tn |
| NZ_FOAL01000016.1 | *Bacteroides thetaiotaomicron* | KPPR-3 | 50034 | 72843 | - | Tn |
| NZ_JH976467.1 | *Parabacteroides johnsonii CL02T12C29* | CL02T12C29 | 152432 | 172162 | - | Tn |
