# Supplementary material for: A Distinct Contractile Injection System Gene Cluster Found in a Majority of Healthy Adult Human Microbiomes
Source: mSystems. 2020 Jul 28;5(4):e00648-20. doi: 10.1128/mSystems.00648-20 (PMC7394362; doi:10.1128/mSystems.00648-20)
Supplement: TABLE S4 [file mSystems.00648-20-st004.docx]

| **Locus Tag (IMG: GOLD Analysis Project ID)** | **KEGG ID** | **Protein ID** | **Gene annotation** | **T4 homolog** | **pADAP homolog** |
| --- | --- | --- | --- | --- | --- |
| Ga0123724_112369 | BcellWH2_03967 | >WP_029427212.1 | DUF4255 | - | Afp16 |
| Ga0123724_112370 | BcellWH2_03968 | >WP_029427211.1 | Hypothetical protein | - | - |
| **Ga0123724_112371** | **BcellWH2_03969** | >WP_029427210.1 | **Sheath1** | **gp18** | **Afp2/3/4** |
| **Ga0123724_112372** | **BcellWH2_03970** | **>WP_029427209.1** | **Sheath2** | **gp18** | **Afp2/3/4** |
| **Ga0123724_112373** | **BcellWH2_03971** | **>WP_007212392.1** | **Tube1** | **gp19** | **Afp1/5** |
| **Ga0123724_112374** | **BcellWH2_03972** | **>WP_007212393.1** | **Tube2** | **gp19** | **Afp1/5** |
| Ga0123724_112375 | BcellWH2_03973 | **>WP_007212394.1** | Hypothetical protein | - | Afp6 |
| Ga0123724_112376 | BcellWH2_03974 | >WP_029427208.1 | LysM | gp6 | Afp7 |
| Ga0123724_112377 | BcellWH2_03975 | >WP_029427207.1 | Spike | gp5 | Afp8 |
| Ga0123724_112378 | BcellWH2_03976 | >WP_029427206.1 | Tip | gp5.4 | Afp10 |
| Ga0123724_112379 | BcellWH2_03977 | >WP_029427205.1 | Baseplate | gp25 | Afp9 |
| Ga0123724_112380 | BcellWH2_03978 | >WP_029427203.1 | Hypothetical protein | - | - |
| **Ga0123724_112381** | **BcellWH2_03979** | **>WP_029427202.1** | **Baseplate** | **gp27** | **Afp11** |
| Ga0123724_112382 | BcellWH2_03980 | >WP_029427201.1 | Hypothetical protein | - | Afp13 |
| Ga0123724_112383 | BcellWH2_03981 | >WP_029427200.1 | Baseplate | gp6 | Afp12 |
| Ga0123724_112384 | BcellWH2_03982 | >WP_029427199.1 | Hypothetical protein | - | Afp14 |
| Ga0123724_112385 | BcellWH2_03983 | >WP_029427198.1 | DUF4157 | - | - |
| Ga0123724_112386 | BcellWH2_03984 | >WP_007215181.1 | FtsH/ATPase | - | Afp15 |
