# Supplementary material for: A Distinct Contractile Injection System Gene Cluster Found in a Majority of Healthy Adult Human Microbiomes
Source: mSystems. 2020 Jul 28;5(4):e00648-20. doi: 10.1128/mSystems.00648-20 (PMC7394362; doi:10.1128/mSystems.00648-20)
Supplement: TABLE S5 [file mSystems.00648-20-st005.docx]

| *.* | **Healthy - IBD** | | **Healthy - Prediabetes** | | **Prediabetes - IBD** | | **Crohn's - colitis** | |
| --- | --- | --- | --- | --- | --- | --- | --- | --- |
|  | **W** | **p-value** | **W** | **p-value** | **W** | **p-value** | **W** | **p-value** |
| **BIS protein count** | 17758 | < 2.2e-16 | 3307 | 0.3716 | 2419.5 | 4.05E-08 | 1144 | 0.5339 |
| **Bacteroidetes abundance** | 14712 | 1.37E-06 | 4652 | 1.99E-06 | 1092 | 0.04941 | 1175 | 0.6817 |
|  | **Alternative hypothesis: true must not equal to 0* | | | |  |  |  |  |
